# Prevalence and associated factors of psychosocial and behavioral problems in Indonesian adolescent students during the COVID-19 pandemic

**DOI:** 10.3389/fped.2022.908384

**Published:** 2022-08-26

**Authors:** Retno Sutomo, Fadhila Pratama Rizqi Ramadhani, Intan Noor Hanifa

**Affiliations:** ^1^Department of Child Health, Faculty of Medicine, Public Health, and Nursing, Universitas Gadjah Mada/Dr. Sardjito Hospital, Yogyakarta, Indonesia; ^2^Faculty of Medicine, Public Health, and Nursing, Universitas Gadjah Mada, Yogyakarta, Indonesia

**Keywords:** COVID-19 pandemic, psychosocial problem, behavioral problem, adolescent, SDQ, screen time, family conflict, family cohesion

## Abstract

**Background:**

The COVID-19 pandemic and the subsequent measures to control it, such as social distancing, school closure, and online learning, put adolescent students at higher risk of psychosocial and behavioral problems (PSBP). The adverse potential is more concerning as the outbreak continues, especially in limited-resource countries, and requires further mitigation.

**Objective:**

To assess the prevalence and factors associated with PSBP in Indonesian adolescent students in the COVID-19 pandemic

**Subject/methods:**

We conducted a cross-sectional study in Yogyakarta Province, Indonesia, involving junior high school students. An anonymous online questionnaire in google form format was used to collect demographic data and the potential variables and screen the PSBP with the Strengths and Difficulties Questionnaire (SDQ). Logistic regression was applied to determine the independent variables.

**Results:**

Six hundred seventy-six subjects participated, including 237 males (35.1%) and 439 females (64.9%). There were 34.6% subjects with PSBP, with a peer-relation problem as the most common one. The multivariable logistic regression showed that subjects with longer screen time duration and more family conflicts were more likely to have PSBP, with an adjusted odds ratio (OR) of 1.5 (95% CI: 1.1–2.1, *p* = 0.025) and 2.4 (95% CI: 1.5–3.8, *p* < 0.001), respectively, whereas whom with better family cohesion are less likely to have the problem with an adjusted OR of 0.4 (95% CI: 0.3–0.6, *p* < 0.001).

**Conclusions:**

There is a high prevalence of PSBP among Indonesian adolescent students during the COVID-19 pandemic. Longer screen time duration and more family conflict are associated with higher prevalence, whereas better family cohesion with lower prevalence of PSBP.

## Introduction

The COVID-19 outbreak constitutes the greatest public health crisis. Since the World Health Organization (WHO) declared a public health emergency of international concern (PHEIC) in January 2020 and then a pandemic in March 2020, it has led to millions of confirmed cases and deaths globally, including in Indonesia. Children with COVID-19 infection show a milder clinical course and better prognosis than adults (1, 2). However, they are more vulnerable to the psychosocial and behavioral impacts of the pandemic. The impacts are particularly concerning for adolescents since they are in a stage of extensive emotional and social development (3). Recent systematic reviews and metaanalysis revealed a higher prevalence of mental problems in adolescents than younger children during this pandemic (4–6).

The Indonesian government started implementing school closure in March 2020 to limit the spread of the infection. The measure puts an additional burden on adolescent students. They have to undergo distance/online learning, spend most of their time in-home, and lack interaction with their peers, increasing their risk of developing psychosocial and behavioral problems (PSBP) (6–9).

Previous studies showed several factors related to the PSBP in adolescents during the COVID-19 pandemic, such as gender (4–6), parental education, family cohesion, domestic violence, and screen exposure (6, 8, 10). There are limited data on this particular issue during the pandemic in Indonesia. A study in the early phase of the pandemic in Indonesia revealed that adolescents were at increased risk for mental health problems (11). With the continuation of the pandemic and school closure policy, the distress related to the pandemic itself and online learning difficulties is progressively accumulating. This study aimed to assess the PSBP and the associated factors in adolescent students approximately after 1 year of the pandemic in Indonesia.

## Methods

### Study design and participants

This cross-sectional study was conducted in Yogyakarta Province of Indonesia in February-May 2021. The participants consisted of junior high school students in the Sleman district of the region. An anonymous online questionnaire in google form format was shared *via* social media or email, facilitated by each classroom teacher. Written informed consent was incorporated in the google form. Participation was entirely voluntary, and only students with whom they and their parents agreed to participate proceeded to fill out the questionnaire. There was no monetary compensation for all participants. All data were kept confidential.

### Instrument

The internet-based questionnaire was used and covered demographic data, such as ages, gender, parental age and education, and the number of family members. It also included several variables potentially associated with PSBP, i.e., screen time duration, screen time for learning or non-learning purposes, and family cohesion and conflicts (before vs. during the pandemic). PSBP was screened using the Strengths and Difficulties Questionnaire (SDQ) adolescent version, included in the online form. SDQ is a widely used tool to screen psychosocial and behavioral problems in children and adolescents. The screening tool has shown good performance in community (12), clinical (13), and school (14) settings. SDQ has been translated into many languages and is available at www.sdqinfo.com. It has also been translated and assessed for its validity and reliability in Indonesian setting (15). It is a brief instrument consisting of 25 items, either positive or negative statements. Each item is scored on a Likert-type scale of 0, 1, or 2 for “not true,” “somewhat true,” or “certainly true,” respectively. The 25 items can be divided into five subscales, including emotional (5 items), conduct (5 items), hyperactivity/inattention (5 items), peer relationship (5 items) problems, and prosocial behavior (5 items). The first four subscales generate total difficulties scores (TDS), consisting of 20 items, whereas the last subscale (5 items of prosocial behaviors) constitutes the strength score. For adolescents aged 11–16, the questionnaire is self-completed. The TDS would range from 0 to 40, and scores of 0–15, 16–19, and 20–40 are classified as normal, borderline, and abnormal. For analysis purposes, the borderline and abnormal groups were merged as an at-risk group and considered as having the PSBP. Each difficulty subscale was also ordered into a normal and at-risk group, respectively: emotional problems (a total score <6 vs. ≥6), hyperactivity behavior (a total score <6 vs. ≥6), peer-relationship problems (a total score <4 vs. ≥4), conduct behavior (a total score <4 vs. ≥4). For the total strength score, the higher the prosocial behavior score (maximum score of 10), the lower the risk for prosocial behavior problems. The total prosocial behavior score >5 was categorized as normal and ≤5 as at-risk group.

### Statistical analysis

Data analyses were performed using statistical software SPSS (IBM Corp., Chicago, IL, USA). Numerical data were expressed as mean and standard deviation (SD) and categorical data as proportion. Bivariate analysis was performed using the Chi-square test to determine the significance and strength of the association between each variable and the PSBP. The potential variables evaluated were gender, screen time duration, screen time utilization, father's and mother's education, family size, cohesion, and conflict. Significant variables in the bivariate analysis were further analyzed by a multivariable logistic regression analysis to determine the variables independently associated with the PSBP. The results of the statistical analysis were expressed as odds ratios (OR) and 95% confidence intervals (95% CI), with a *p*-value < 0.05 was considered statistically significant.

### Ethical approval

This study has been approved by The Medical and Health Research Ethics Committee of the Faculty of Medicine, Public Health, and Nursing, Universitas Gadjah Mada, Yogyakarta, Indonesia.

## Results

There were 676 students enrolled in this study, consisting of 237 males (35.1%) and 439 females (64.9%). The mean age was 13.6 years old. Most parents (64.4% fathers and 62.6% mothers) have 12 years or more of formal education. The mean screen time during the pandemic significantly increased compared to that before the pandemic (4.7 vs. 7.9 h per day). The proportion of subjects with longer screen time (>mean) is slightly higher (52.1%). More subjects used longer screen time for learning purposes (56.8%); however, a significant proportion of subjects did it for non-learning purposes (43.2%). Most participants reported having better family cohesion (64.9%) and less family conflicts (87.3%) during the pandemic. There were 34.6% subjects at risk for PSBP, with a peer-relation problem as the most prevalent (46.7%). All students underwent distant/online learning during the period of study. The detailed characteristics of the participants were shown in Table 1.

**Table 1:** Baseline characteristics of subjects (*N* = 676)^*^.

**Characteristics**	**Mean (SD) or n (%)**
Age (years)	13.9 (0.8)
Gender	
Boy	237 (35.1)
Girl	439 (64.9)
Father's age (years)	46.7 (0.2)
Mother's age (years)	43.3 (0.2)
Father's education level	
Lower education (≤ 12 years education)	239 (35.4)
Higher education (>12 years education)	437 (64.6)
Mother's education level	
Lower education (≤ 12 years education)	253 (37.4)
Higher education (>12 years education)	423 (62.6)
Number of family members	
Smaller family (4 or less)	348 (51.5)
Larger family (5 or more)	328 (48.5)
Screen time (hours per day)	
Before the pandemic	4.8 (3.3)
During the pandemic	7.9 (3.9)
Screen time during the pandemic (hours per day)	
Shorter screen time (≤ mean)	324 (47.9%)
Longer screen time (> mean)	352 (52.1%)
Screen time use	
Longer for learning purpose	384 (56.8)
Longer for non-learning purpose	292 (43.2)
Family cohesion during the pandemic	
More cohesive	439 (64.9)
Less cohesive	237 (35.1)
Family conflict during the pandemic	
More conflict	86 (12.7)
Less conflict	590 (87.3)
Psychosocial and behavioral problems	
Total difficulty score (TDS)	
Normal	449 (66.4)
At-risk	227 (34.6)
Emotional problem	
Normal	485 (71.7)
At-risk	191 (28.3)
Conduct behavior	
Normal	531 (78.6)
At-risk	145 (21.4)
Hyperactivity behavior	
Normal	531 (78.6)
At-risk	145 (21.4)
Peer-relation problem	
Normal	360 (53.3)
At-risk	316 (46.7)
Prosocial behavior problem
Normal	612 (90.5)
At-risk	64 (9.5)

* Values are mean (standard deviation) for numerical variables or numbers (percentages) for categorical variables.

Bivariate analysis using the Chi-square test showed that five predictors, i.e., female gender, larger family, more family conflict, better family cohesion, and longer screen time, were significantly associated with PSBP. On subsequent multivariable logistic regression analysis (Table 2), only three predictors were independently associated with the prevalence of PSBP, i.e., longer screen time and more family conflicts were associated with higher whereas better family cohesion with lower prevalence of PSBP.

**Table 2:** Analysis of variables associated with PSBP.

**Variables**	**PSBP (227)**	**Normal (449)**	**Bivariate analysis**		**Multivariable analysis**	
	***n* (%)**	***n* (%)**	**Unadjusted OR (95% CI)**	***P***	**Adjusted OR (95% CI)**	***P***
Female gender	159 (70.0)	280 (62.4)	1.4 (1.1–2.0)	0.05	1.3 (0.9–1.8)	0.16
Father's lower education	94 (41.4)	155 (34.5)	1.1 (0.8–1.6)	0.52		
Mother's lower education	84 (37.0)	169 (37.6)	1.0 (0.7–1.4)	0.87		
Longer screen time (> mean)	134 (59.0)	218 (48.6)	1.53 (1.11–2.11)	0.01	1.5 (1.1–2.1)	0.025
Longer screen time for non-learning purpose	104 (45.8)	188 (41.9)	1.2 (0.9–1.6)	0.33		
Larger family	125 (55.1)	203 (45.2)	1.5 (1.1–2.1)	0.02	1.4 (1.0–1.9)	0.07
More family conflict	48 (21.1)	38 (84.6)	2.9 (1.8–4.6)	<0.001	2.4 (1.5–3.8)	<0.001
Better family cohesion	118 (51.5)	321 (71.5)	0.5 (0.4–0.7)	<0.001	0.4 (0.3–0.6)	<0.001

PSBP, Psychosocial and behavioral problem; OR, Odds ratio; CI, Confidence interval.

## Discussion

This study found that after ~1-year-pandemic in Indonesia, 34.6% of adolescent students experienced PSBP, with peer relationships problems as the most prevalent. Longer screen time and more family conflicts are risk factors, whereas better family cohesion is protective against the problems.

The prevalence observed in this study is similar to that estimated in a recent metanalysis (4), which showed that 34.4 and 29.1% adolescents suffered depression and anxiety, respectively, during the pandemic. However, it is exceedingly higher than the finding from another study in Indonesia carried out within 1–2 months after the pandemic, i.e., 14.2% (11). Both studies are comparable in terms of using the same screening tool (SDQ), classifying the PSBP into “normal” or “at-risk,” and covering subjects of similar ages. This finding suggests an accumulation of adverse impacts with the pandemic's extension. Adverse factors act chronically and cumulatively in invoking psychological and behavioral problems in children. The longer and the more adversities children experience, the worse the problems (16–18). The PSBP can be associated with pandemic-related stress, such as uncertainty about when the pandemic will end, fear of infection affecting themselves, family members, or friends. In addition, school closure is disruptive for children and adolescents. For them, schools are beyond a mere educational hub (7, 19). They spend a large portion of their lives at school. Their relationships with teachers and peers and the school atmospheres provide a support system, which is essential for their psychological, emotional, and behavioral wellbeing. There is also fear about compromised academic achievement from school closure, adding more unfavorable factors for mental health problems (20, 21). Furthermore, school closure makes students undergo full online learning, which poses many challenges, especially in low-middle income countries, such as Indonesia. Many students may have no gadgets required for facilitating online learning. Internet connection is not always available in all areas, possibly unaffordable for many households, and the connection quality is often unstable. However, it should be noted that there is no unexposed-to-pandemic group in this study. In addition, we have no comparable data on the prevalence of the PSBP in Indonesia (screened by SDQ in a similar group of subjects) before the pandemic period. Thus, there is a possibility that the risk factors are different in nature or magnitude.

This study showed peer relationships as the most common problem. It is consistent with the previous study (11), indicating that peer relationships are crucial for adolescents during their developmental stage.

Our study showed that screen time, family conflict, and better family cohesion were independently associated with PSBP. Subjects with longer screen time and more conflicts in the family are 1.5 and 2.4 times, respectively, more likely to have PSBP. Meanwhile, those with better family cohesion during the pandemic are 2.5 times less likely to have the problems.

The mean screen time recorded in this study was 7.9 h, significantly longer than before the pandemic (4.8 h). Thus, even before the pandemic, it has exceeded the recommendation on the maximum duration of screen exposure (22). Unsurprisingly, the pandemic has increased screen time (7, 23–25) for many reasons and purposes. Adolescents use it not only for online learning due to school closures but also for entertainment and social interaction due to home confinement (26). Screen time during the pandemic is like a double-edged sword. Excessive screen time may adversely affect adolescents' wellbeing. A study in Italian adolescents (27) found more problematic social media use during the pandemic and it was associated with more emotional and behavioral symptoms. An overabundance of information about the pandemic on the internet and social media may be harmful to mental health (19). However, screen time during home confinement may also be helpful in maintaining social relationships with peers and relatives, allowing self-expression, and coping with boredom, loneliness, and anxiety. In that regard, social media use can be favorable in preventing adolescents from psychosocial problems (28, 29). There is a view (30) that the impact of screen time for adolescents who are physically distancing depends not only on time spent online but also on whether it is connection promoting (i.e., active and communicative) or non-connection promoting (i.e., passive). This study found that 43.2% of subjects spent screen time for non-learning purposes but no difference in PSBP between the group with longer screen time for learning and those for non-learning purposes. Unfortunately, our study did not specify the “non-learning purposes,” whether they were connection promoting or non-connection promoting activities.

The majority of adolescents in this study have less family conflicts during the pandemic, but it should be noted that those with more family conflicts are prone to have PSBP. Many adolescents may experience increased conflicts due to elevated distress both in children and parents (31). The parents may have additional burdens related to a financial stressor or employment loss (32). Previous studies have shown that family conflict adversely influences adolescents' mental health, both before (33, 34) and during (35, 36) the COVID-19 pandemic.

Our data showed that 64.9% of adolescents perceived having better family cohesion, and they are less likely to have PSBP. This data is consistent with previous studies showing lower internalizing or externalizing problems among adolescents in cohesive families (37). Some adolescents may benefit from more intense interaction with parents and siblings during prolonged home confinement (31). Social distancing may strengthen the sense of community and family attachment, especially in a culture of collectivism, such as in Indonesian society. Good family cohesion increases social support, which strengthens resilience (38–40) and impacts favorably on mental health outcomes (6). Thus, better family cohesion may protect adolescents against distress during the COVID-19 crisis.

This study adds valuable information regarding the high prevalence of PSBP in adolescents and the associated factors around 1 year after the pandemic hit Indonesia. However, this study has some limitations. First, this study did not explore the specific sources of the distress, including the pandemic itself (e.g., knowledge and perception), the school closure, the online learning with the related problems encountered), or responses related to local cultural values. Second, the subjects of this study are limited to one district in Yogyakarta Province, which possibly limits the generalizability of the findings. This disadvantage, however, may be accounted for since at the time of this study, the Indonesian government implemented the policy of full school closure, and all students in Indonesia generally encountered similar problems related to online/distance learning. Finally, the cross-sectional design makes it is unable to infer the causal relationship.

The findings of this study suggest the importance of screening and mitigating the PSBP in adolescent students during the pandemic. Identifying factors related to those problems would be necessary for planning resources, designing interventions, and developing relevant policies. The implementation of school closure and the subsequent distance/online learning should be balanced with measures to minimize the adverse impact on their wellbeing. The schools, teachers, and adolescents should be more involved in formulating the measures and policy.

## Conclusion

The prevalence of psychosocial and behavioral problems among Indonesian adolescents is remarkably high during 1 year period pandemic. Screen time duration, family conflict, and family cohesion are independently and significantly associated with the problems.

## Data availability statement

The raw data supporting the conclusions of this article will be made available by the authors, without undue reservation.

## Ethics statement

The studies involving human participants were reviewed and approved by the Medical and Health Research Ethics Committee (MHREC) Faculty of Medicine, Public Health, and Nursing, Universitas Gadjah Mada, Indonesia. Written informed consent to participate in this study was provided by the participants' legal guardian/next of kin.

## Author contributions

RS conceptualized and designed the study, identified the statistical methods, validated the statistical analysis results, and drafted and finalized the manuscript. FR searched the relevant literature, collected data, and drafted the manuscript. IH collected data, organized the database, and performed the statistical analysis. All authors contributed to the manuscript revision and approved the submitted version.

## Conflict of interest

The authors declare that the research was conducted in the absence of any commercial or financial relationships that could be construed as a potential conflict of interest.

## Publisher's note

All claims expressed in this article are solely those of the authors and do not necessarily represent those of their affiliated organizations, or those of the publisher, the editors and the reviewers. Any product that may be evaluated in this article, or claim that may be made by its manufacturer, is not guaranteed or endorsed by the publisher.
